# Aptamers Against COVID-19: An Untested Opportunity

**DOI:** 10.2174/1389557522666220112094951

**Published:** 2022

**Authors:** Annekathrin Haberland, Johannes Müller

**Affiliations:** 1 Berlin Cures GmbH, Robert-Rössle-Str. 10, 13125 Berlin, Germany;; 2 Berlin Cures GmbH, Knesebeckstr. 59-61, 10719 Berlin, Germany

**Keywords:** Aptamer, BC 007, COVID-19, long-COVID-19, multiple modes-of-action, SARS-CoV-2

## Abstract

Given the lack of success in the development of effective drugs to treat COVID-19, which show “game-changing” potential, it is necessary to explore drugs with different modes of action. Single mode-of-action drugs have not been succeeded in curing COVID-19, which is a highly complex disease. This is the case for direct antivirals and anti-inflammatory drugs, both of which treat different phases of the disease. Aptamers are molecules that deliver different modes of action, allowing their effects to be bundled, which, when combined, support their therapeutic efficacy.

In this minireview, we summarise the current activities in the development of aptamers for the treatment of COVID-19 and long-COVID. A special emphasis is placed on the capability of their multiple modes of action, which is a promising approach for treating complex diseases such as COVID-19.

## 1 INTRODUCTION

Medical care for patients who are severely ill with coronavirus disease 2019 (COVID-19) has significantly improved since the beginning of the pandemic in spring 2020. The constant attention of clinicians and nurses, who have gained a lot of experience in dealing with COVID-19 patients, has led to the continuous improvement and optimisation of therapy [1].

However, as far as targeted support with drugs is concerned, there has been little significant progress. Unfortunately, the “universal pill” against COVID-19 has not yet been found.

Science has not yet fully grasped the complexity of the disease, and all aspects of the pathogenesis cannot be covered with one active principle. Severely ill patients undergo several individual phases of the disease, from acute infection to an inflammatory phase, which often results in lung injury and/or multiorgan failure. This has been well described by Suman Rohilla, who collected all pathological events underlying COVID-19 disease in a review focused on the design of therapeutic strategies to treat severe cases [2]. Hu *et al.* demonstrated the persistence of an active virus in the gastrointestinal tract after being cleared by the respiratory tract [3], which could explain why these phases can merge into one another.

Many therapeutic principles are highly specific to their target, which is the virus in this case, including targeted antiviral drugs and therapeutic anti-SARS-CoV-2 monoclonal antibodies. Besides the viral phase, several different drugs have been tested against the other phases of the disease, primarily the inflammatory phase. Success has been limited, with only dexamethasone showing significant improvement, and only for patients on ventilators in an advanced state of disease [4]. Also, “*using the right dose, at the right time, in the right patient* “ seemed to be a critical point for the dexamethasone success, as cited by Shabalin *et al.* [5], from the recovery trial. This statement prompted Shabalin and his collaborators to investigate the molecular mechanism on which these effects in COVID-19 patients might be based. The drug’s vascular transport connected with its albumin binding, which itself is easily influenced by many factors, seemed to play an essential role in its availability and effectivity and should be considered for future individual dosing regimen adjustments [5].

With respect to direct antivirals, most recently, Molnupiravir became a new hope [6].

When developing a drug, high specificity is usually aimed for a “single-target specificity approach” [7], which is not always possible. Otherwise, side effects would be rare, which is not the case. With classical drug development, polypharmacology has become a new paradigm for addressing multifactorial complex diseases [7].

In the case of COVID-19, such a rethinking process should be considered. Drugs that present the option to develop multiple modes of action (“multitarget drug discovery” [7]), all supporting the therapy goal, should even be prioritised.

One promising treatment is aptamers, which are small, single-stranded RNA or DNA sequences that bind targets based on their structure, similar to antibodies. Therefore, they are often referred to as “chemical antibodies” [8]. Most aptamers are empirically selected against the target of interest. Although they are supposed to be highly specific to their target, there is also information that a particular 3D structure opens up the possibility of binding other molecules with more or less affinity. This is the case for the G-quadruplex structure [9]. This characteristic opens the possibility to bundle several mode-of-action principles supporting the therapeutic effect in the same aptamer. This may be a promising approach for complex diseases such as COVID-19. Most recently, an *in silico* approach identified a G-quadruplex sequence as a binder of the RBD sequence of the spike protein of SARS-CoV-2 [10].

This brief review will provide an overview of the current activities in this field, with a special focus on the multiple mode-of-action capabilities of anti-COVID-19 aptamers. This makes this review very different from recent reviews, which have focused on aptamer technology in the combat of COVID-19, such as the one by Dzuvor *et al.* [11], who focused on diagnosis only. Torabi *et al.* [12] already mentioned both diagnosis and therapy, but the paper was written and published in the early stages of the pandemic crisis, in spring/summer 2020, with no concrete developments insight.

## 2 APTAMERS AS ANTIVIRALS - SOME HISTORICAL ASPECTS

A variety of aptamers have been developed to combat viral infections and as diagnostic tools over the last few decades. Kim and Lee [13] and Kürger *et al.* [14] recently published comprehensive overviews of these activities. There are examples of aptamers that have already shown *in vivo* efficacy for treating influenza in an animal model [15].

With respect to HIV therapy, aptamer options that were comprehensively summarized by Bala *et al.* [16], aptamer T30177, an inhibitor of the virus integrase, was the first integrase inhibitor tested in clinical trials (Zintevir™, Aronex Pharmaceuticals) [17, 18]. To our knowledge, however, no aptamer therapies have reached the market for this indication.

To date, the pressure for success has clearly not been high enough. This can be compared to a different oligonucleotide drug, mRNA vaccines, which had been under development for decades prior to the COVID-19 pandemic [19]. The urgency of the COVID-19 pandemic provided the pressure to succeed, along with the necessary financial basis to accelerate the development. Europe supported the vaccine research with “more than €1 billion…from Horizon 2020…bringing together “the total amount of support to over €30 billion” (https://ec.europa.eu/info/research-and-innovation/research-area/health-research-and-innovation/coronavirus-research-and-innovation/vaccines_en).

COVID-19 drug development has not received the same attention as vaccines, at least in Europe, especially in terms of financial support (https://ec.europa.eu/info/live-work-travel-eu/coronavirus-response/public-health/treatments-covid-19_en). This is unfortunate, as therapeutic opportunities could be lost.

## 3 APTAMERS AS ANTI-SARS-COV-2 AGENTS AND COVID-19 THERAPEUTICS

### 3.1 SP6

The only aptamer project supported by the German Ministry of Education and Research (BMBF) in 2020/21 was focused on the selection of spike protein-binding ssDNA aptamers to inhibit the interaction of the virus with the ACE2 receptor. The first effective ssDNA sequences were published in March 2021 by Schmitz and colleagues, of which SP6 was identified as the most effective aptamer [20]. However, their lead candidate did not inhibit the interaction of the spike protein with the ACE2 protein, but it still inhibited viral growth. The mechanism by which SP6 inhibits viral infection is still unknown, with the authors stating: “*A remarkable and unexpected feature of SP6 is that its inhibitory effect does not result from interfering with the interaction of CoV2-S with ACE2.*”

Even though the exact mode of action of SP6 is not known, this is considered the starting point for its continued development as a COVID-19 therapeutic (https://www.scinexx.de/news/medizin/corona-neuartiger-hemmstoff-identifiziert/).

The observation that there is likely to be a different mode of action implies that this aptamer binds and inhibits viral growth by targeting a different protein or range of proteins.

### 3.2 BC 007

Aptamer BC 007, which was originally developed as a thrombin inhibitor [21], but failed because its effect was too moderate, is an effective neutraliser of certain pathogenic functionally active autoantibodies [22-24]. It is currently being tested in a clinical phase 2a trial (NCT04192214) as a causal heart failure therapeutic for the neutralisation of pathogenic functionally active autoantibodies against the β1-adrenoceptor. More recently, this aptamer has also demonstrated specific binding to two crucial growth proteins of the SARS-CoV-2 virus, the spike protein and the RNA-dependent RNA polymerase [25]. Although currently untested, binding to other viral sequences is highly probable. This might be the case for the helicase enzyme, which has previously been the target for aptamer selections [26], or the nucleocapsid protein [27]. Furthermore, aptamers against SARS-CoV-1 have been previously reported [26, 28].

The multiple binding sites of BC 007 for crucial growth SARS-CoV-2 proteins provide this aptamer a great advantage over the binding principle of therapeutic monoclonal antibodies. This increases the chance of therapeutic success, and it may even lower the risk of escape mutations or a loss of efficacy in the case of virus variants.

Importantly, some coronaviruses, especially aggressive ones such as SARS-CoV-1 and -2, carry the G-quadruplex DNA or RNA-binding-specific protein sequence, which is rare in the viral world, in its largest non-structural protein complex Nsp3. This quadruplex oligonucleotide binding-specific protein sequence is called the ***SARS-unique domain*** (SUD) [29, 30]. Aptamer BC 007 forms a G-quadruplex in the presence of certain ions [31], but also when binding target peptides and proteins [25, 32]. This is also the case for SP6. At SP6, several G-quadruple options were identified using a publicly accessible QGRS mapper (for the identification of G-quadruple options in nucleotide sequences (http://bioinformatics.ramapo.edu/QGRS/analyze.php; Ramapo College of New Jersey, Department of Bioinformatics, USA; [33]). This aptamer might inhibit SARS-CoV-2 growth by binding to the SUD.

### 3.3 AS1411

SARS-unique domain binding is also probable when looking at AS1411. AS1411 is a G-quadruplex aptamer of the sequence 5’-GGTGGTGGTGGTTGTGGTGGTGGTGG, which differs from that of BC 007 (5'-GGTTGGTGTGGTTGG). This aptamer is currently being considered for COVID-19 treatment through a repurposing program (https://sciencebusiness.technewslit.com/?p=39297), and it received the “go ahead” from the FDA for clinical testing at the end of 2020 (https://www.prnewswire.com/news-releases/qualigen-therapeutics-receives-positive-pre-ind-response-from-fda-for-the-clinical-development-of-as1411-as-a-treatment-for-covid-19-301161655.html).

In its original indication as cancer therapeutic, AS1411 targets nucleolin [34]. Nucleolin is not only an essential factor in cancer cell growth [35], but it is also involved in RNA virus infectivity [36]. Therefore, a therapeutic effect has been proposed for COVID-19 (https://www.prnewswire.com/news-releases/qualigen-therapeutics-receives-positive-pre-ind-response-from-fda-for-the-clinical-development-of-as1411-as-a-treatment-for-covid-19-301161655.html).

However, AS1411 also presents other effects, including an effect on nuclear factor-kappaB (NF-κB) essential modulator (NEMO), which is a regulatory subunit of the inhibitor of kappaB (IκB) kinase (IKK) complex (also called IKKgamma) [37]. This effect is thought to be of additional benefit for cancer therapy, and it may also benefit COVID-19 therapy. SARS-CoV-2 can activate NF-κB, which causes subsequent neurological complications, as outlined recently in a review by Bhandari and coworkers [38]. Interruption of such a process should be beneficial in any case.

The ability of aptamers to be able to attack multiple targets [39] will open up the normally small window of therapeutic intervention. For COVID-19, this might represent a great advantage compared to specific drugs.

## 4 FURTHER APTAMERS AGAINST SARS-COV-2

Further reports about the successful selection of aptamers against SARS-CoV-2 proteins include **aptamer-1**, **-2**, and -**6**, selected against the spike protein/receptor-binding domain (S/RBD) in March 2020 [40]. The sequences are as follows: 5’-TCGAGTGGCTTGTTTGTAATGTAGGGTTCCGGTCGT-GGGT for aptamer-1, 5’-ATTACCGATGGCTTGT-TTGTAATGTAGGGTTCCGTCGGAT for aptamer-2, and 5’-GGGCTTGGGTTGGGAATAAGGATGTGGGAGGCG--GCGAACA for aptamer-6. Only the last one is theoretically able to form a G-quadruplex structure. However, this was the only aptamer that did not show virus neutralisation ability, even though the binding was confirmed with K_D_ values similar to aptamer-1 and -2. The faster K_a_ and K_d_ kinetics of aptamer-6 compared to aptamer-1 and -2 will result in a lower maximal binding capacity (B_max_), which is thought to be a possible reason for its inability to interfere with S/RBD ACE2-receptor binding [40].

**CoV2-RBD-aptamers 1–6,** also selected against the spike protein, were reported by Song and coworkers [41, 42]. There are length-optimised versions of these aptamers. For example, CoV2-RBD aptamer-1C is a 51mer three-hairpin-structure aptamer of the following sequence: 5’-CAGCACCGACCTTGTGCTTTGGGAGTGCTGGTCCA-AGGGCGTTAATGGACA, which is theoretically able to form the G-quadruplex structure [33]. CoV2-RBD-4C is a 67mer aptamer of the sequence 5‘-ATCCAGAGTGACGC-AGCATTTCATCGGGTCCAAAAGGGGCTGCTCGGGATTGCGGATATGGACACGT, which also contains a quadruplex-forming G-rich sequence. Both of these aptamers show a high binding affinity towards the RBD protein of the virus [41].

Aptamer CoV2-RBD-1C, developed by Song *et al.* [41], has recently been reported to bind not only to its selection target, the recombinant protein, as described by Song and coworkers but also to the native SARS-CoV-2 virus, as reported by Singh *et al.* [43]. These authors successfully used this sequence to develop a highly sensitive aptamer-based diagnostic test. Therefore, the aptamer selected and reported by Song *et al.* has already been successfully exploited in SARS-CoV-2 diagnostics.

Another development was reported by Sun *et al.* [42]. Firstly, they combined their selection procedure with modelling aspects to optimise and accelerate development; then, they modified their best hit for binding optimisation and stabilisation. This resulted in **CoV2-6C3,** a possible G-quadruple-forming aptamer of the following sequence: 5’-CGCAGCACCCAAGAACAAGGACTGCTTAGGATTGCGATAGGTTCGG, which showed the best S-ACE2 binding inhibition effect in their experimental set-up. This was the starting point for the subsequent development of a circular bivalent construct that was able to bypass exonuclease susceptibility. This resulted in greatly enhanced serum stability and increased binding affinity. This product was demonstrated to be an effective inhibitor of SARS-CoV-2 uptake by cells (IC_50_ = 0.42 nM), and it reduced the amount of viral RNA in cells by about 85% [42].

Most recently, aptamer **nCoV-S1-Apt1** has been described to bind the RBD with high affinity. According to the authors of this study, nCoV-S1-Apt1 “*displayed a dose-dependent inhibitory profile on pseudovirus infection*” [44].

## 5 FURTHER EFFECTS OF APTAMERS

As discussed previously, BC 007 neutralises pathogenic functionally active autoantibodies against G-protein coupled receptors [22]. Furthermore, this effect not only occurs *in vitro* in a laboratory setting but also in humans, probands [23], and patients (NCT04192214). Such pathogenic autoantibodies have already been observed in patients with COVID-19, especially those who suffer from long-lasting symptoms [45, 46]. BC 007 should be able to neutralise these autoantibodies, which has already successfully been seen in a named patient program [47].

However, other functionally active autoantibodies are also thought to be involved in disease persistency [48] and severity [49].

The anticoagulation effect of BC 007, while only moderate and only in parallel to the infusion [24], has an additional positive effect given that micro thromboembolism is an additional threat in COVID-19 patients [50]. To solve this problem, heparin administration has been suggested for hospitalised patients. However, evidence from randomised blinded trials is still missing. Randomised (blinded or not) clinical trials testing the heparin type and dose are currently going on [51]. Data from open studies testing prophylactic *versus* higher doses of low-molecular-weight heparin are promising for the prophylactic dose [52].

However, Liu *et al.* reported that “Heparin-induced thrombocytopenia” (HIT) “is associated with a high risk of mortality in critical COVID-19 patients receiving heparin-involved treatment” [53]. These authors observed that, in a high percentage of their investigated patients, “HIT occurred not only in patients with heparin exposure, such as CRRT” (Continuous renal replacement therapy) “but also in heparin-naïve patients, suggesting that spontaneous HIT may occur in COVID-19.” They came to a conclusion that “Anti-heparin-PF4 antibodies are induced in critical COVID-19 patients, resulting in a progressive platelet decrease. Exposure to a high dose of heparin may trigger further severe thrombocytopenia with a fatal outcome. An alternative anticoagulant other than heparin should be used to treat COVID-19 patients in critical condition.” This has also been supported by observations by other groups. Daviet *et al.* [54] reported an incidence of HIT of 8% in their hospitalised COVID-19 patients, which is clearly higher than that usually observed in COVID-19-free critically ill patients.

Such antidrug reactions are not expected if non-modified aptamers are used as anticoagulatory drugs because non-modified oligonucleotides are not immunogenic (Table **1**) [55].

## 6 DISCUSSION

Aptamers have been suggested for COVID-19 therapy; however, to our knowledge, no aptamers have been tested for COVID-19 efficacy in the clinic so far.

A common characteristic of all these advanced aptamers seems to be that they do not only bind one specific aptatope (epitope for aptamers) but instead target different proteins on the virus, in addition to the host cell. If the combined effects of an aptamer support a therapeutic effect, this could represent a promising solution to combat the disease.

Aptamers that are already advanced in their development are BC 007 and AS1411. Both aptamers have successfully passed phase 1 clinical tests [24], and they are currently in or completed phase 2 tests [56], respectively. Both show excellent safety profiles and good safety and tolerability in very ill patients, who would be the target patients with respect to COVID-19 disease [24, 56]. AS1411 has already received permission from the FDA to carry on with an efficacy trial for COVID (https://www.prnewswire.com/news-releases/qualigen-therapeutics-receives-positive-pre-ind-response-from-fda-for-the-clinical-development-of-as1411-as-a-treatment-for-covid-19-301161655.html).

With BC 007, the developer will prioritize a possible long-COVID therapy [47] without losing sight of the treatment of the acute phase. One might argue that because the aptamer reported by the Schmitz group [20], SP6, was specifically designed for COVID-19, in contrast to BC 007 and AS1411, it might function better. However, the newly and specifically selected aptamer functions differently than expected, with a completely different mode of action than it was designed for. Regarding its mode of action, it cannot be excluded that it may behave similarly to the other more evolved aptamers, BC 007 or AS1411. It is still a different sequence. Supporting only one group or method will not necessarily result in success. This is a lesson we have already learned from SARS-CoV-2 vaccine development.

## CONCLUSION

Aptamers combine several properties that make them perfect candidates as potential COVID-19 therapies. In this way, aptamers could be well suited to the complexity of this disease.

The main advantage of multi-target capable aptamers over highly specific drugs is their combinatorial multiple mode-of-action capabilities.

## Figures and Tables

**Table 1:** Overview of aptamers for COVID-19 treatment.

**No.**	**Code Name**	**Type**	**Target**	**Length**	**Sequence**	**Repurpose**	**State of Clinical Development, Original Indication [Ref]**	**Refs.**	**State of Development, COVID-19 /Long-COVD**	**Refs.**
1	AS1411 (QN-165)	ssDNA	Nucleolin	26	GGTGGTGGTGGTTGTGGTGGTGGTGG	yes	Phase 2	[56]	IND filed	-
			NF-kB							
2	BC007	ssDNA	GPCR-AABs	15	GGTTGGTGTGGTTGG	yes	Phase 1	[24]	Preclinical/named patient program	[47]
			Thrombin				Phase 2a (ongoing)	(NCT04192214)		
			SARS-CoV-2 RBD				-	-	Preclinical	[25]
			RdRP					-	Preclinical	[25]
3	SP6	ssDNA	Unknown (selected against spike protein)	41	CCCATGGTAGGTATTGCTTGGTAGGGATAGTGGGCTTGATG	no	n.a	-	Preclinical	[20]
4	Aptamer-1	ssDNA	SARS-CoV-2 RBD -	40	TCGAGTGGCTTGTTTGTAATGTAGGGTTCCGGTCGTGGGT	no	n.a	-	Preclinical	[40]
5	Aptamer-2	ssDNA	SARS-CoV-2 RBD	40	ATTACCGATGGCTTGTTTGTAATGTAGGGTTCCGTCGGAT	no	n.a	-	Preclinical	[40]
6	CoV2-RBD-aptamers 1C	ssDNA	SARS-CoV-2 RBD	51	CAGCACCGACCTTGTGCTTTGGGAGTGCTGGTCCAAGGGCGTTAATGGACA	no	n.a	-	Preclinical	[41]
7	CoV2-RBD-aptamers 4C	ssDNA	SARS-CoV-2 RBD	67	ATCCAGAGTGACGCAGCATTTCATCGGGTCCAAAAGGGGCTGCTCGGGATTGCGGATATGGACACGT	no	n.a	-	Preclinical	[41]
8	CoV2-6C3	ssDNA	SARS-CoV-2 RBD	46	CGCAGCACCCAAGAACAAGGACTGCTTAGGATTGCGATAGGTTCGG	no	n.a	-	Preclinical	[42]
9	nCoV-S1-Apt1	ssDNA	SARS-CoV-2 RBD	81	n.a.	no	n.a	-	Preclinical	[44]

ssDNA = single stranded DNA, RBD = receptor binding domain, n.a. = not available.
